# The effect of short-term fallowing on the microbial communities in forest soil cultivated with ginseng: Preliminary research

**DOI:** 10.7717/peerj.14758

**Published:** 2023-01-31

**Authors:** Yuqing Li, Feyisike Gbolayori Jones, Bing Zhang, Juntao Cui, Wei Zhang

**Affiliations:** 1 Jilin Agricultural University, College of Resources and Environment, Changchun, Jilin, China; 2 Changchun Polytechnic, Department of Modern Agricultural Technology, Changchun, Jilin, China

**Keywords:** Ginseng, Fallow management, Microbial community, Fungal pathogen

## Abstract

**Background:**

Continuous cultivation of ginseng crops in fixed plots can lead to disease outbreaks, yield losses and replanting failures. Fallow periods can help restore soil health and increase the sustainability of agricultural systems; however, taking land out of production for extended periods is often not feasible. Short-term fallow periods could restore soil health, but few studies have examined the effects of short-term fallow treatment on the health of soil in ginseng fields.

**Methods:**

In this preliminary study, we used metagenomic analysis to assess changes in the abundance of major ginseng pathogens and soil health overall following a short-term fallow period in a region in the Changbai Mountains. A sample from a forest plot (Hx0ks), was compared to a sample from a field where ginseng was previously cultivated and then had been left fallow for two years (Hx2), and a sample from a field that had been fallow for two years and was subsequently replanted with ginseng (Clsd).

**Results:**

Soil that was fallow for two years, and then replanted with ginseng, showed reduced nutrient content and lower diversity of soil bacterial and fungal communities than soil that remained fallow. *Candidatus Solibacter* (5%) and *Rhizomicrobium* (3%) were the most abudant bacterial genera in Hx2. *Rhizomicrobium* (4%) and *Gemmatimonas* (3%) were the most abundant bacterial genera in Clsd. *Mortierella* (22%) and *Peziza* (12%) dominated the fungal community in Hx2. *Lecanicillium* (38%) and *Mortierella* (13%) dominated the fungal community in Clsd. Fallow periods also increased the functional diversity of soil as predicted by PICRUSt and decreased the relative abundance of the pathogenic fungi.

**Conclusions:**

Preliminary findings were consistent with the hypothesis that fallow management in ginseng cultivation can improve soil microbial community structure and function and reduces the number of plant pathogens; however, testing this hypothesis will require replicated plots.

## Introduction

Ginseng (*Panax ginseng*) is a slow-growing herb of the Araliaceae family that is an important medicinal plant in northeast Asia (Suh et al., 2011). Ginseng in cultivation requires four to six years to develop the best shape, root quality and ginsenoside content (Cho et al., 2007; Farh et al., 2018). This long growth cycle increases the abundance of fungal pathogens (Cho et al., 1995). Continuous cultivation of ginseng can degrade soil quality, increase the abundance of plant pathogens and change the composition and diversity of soil microbial communities (Dong et al., 2018c; Li et al., 2020). Microbial communities in ginseng cultivated soil are particularly sensitive to continuous cropping patterns (Dong et al., 2017). These changes are associated with a series of replanting problems, such as poor seedling growth, high disease incidence rate, and decreased yield (Chen et al., 2012). Typically, the survival rate of ginseng seedlings cultivated in the same area was less than 25% and approximately 75% of the crops are infected with various pathogens (Wu et al., 2008). For example, Liu et al. (2021) found that soil pH decreased during ginseng cultivation, and the content of soil salt, ${\mathrm{NH}}_{4}^{+}$-N and ${\mathrm{NO}}_{3}^{-}$-N increased, which led to changes in the composition and diversity of soil bacterial and fungal. Moreover, continuous cropping soil of ginseng significantly changes the soil carbon substrate metabolic spectrum; pathogenic fungi gradually became the dominant flora (Fei, Lin & Chunga, 2021; Ying et al., 2012). These pathogens of ginseng include *Ilyonectria destructans, Cylindrocarpon destructans, Brotrytis cinerea, Fusarium solani, Alternaria panax*, and *Phytophthora cactorum* (Farh et al., 2018; Zheng et al., 2017). Infected ginseng roots cannot be processed or sold (Guan et al., 2020).

The Changbai mountain range in China accounts for 60%–70% of the global production of ginseng roots (Wang et al., 2019). Moist, dark conditions and fertile soil in these mountains are ideal for ginseng cultivation (Zhang et al., 2008). This has led to deforestation of this mountain range and depleted stocks of wild ginseng and degrades limited land resources (Dong et al., 2018b; Zhao et al., 2017). Fallow periods can restore degraded farmland (Manalil, Riethmuller & Flower, 2014), in terms of soil nutrients and microbial diversity (Siddiqi & Im, 2016; Unger & Howell, 2000); however, long-term fallow can cause significant economic losses. Therefore, using short-term fallow to restore soil quality of cultivated land is recommended (Feng et al., 2005; Wang, Shen & Zhang, 2014). However, little is known about whether a short fallow period can eliminate the obstacles to ginseng continuous cropping.

In this study, we compared three soil samples collected in the Changbai Mountains: forest soil, two-year fallow soil, and replanted soil in physicochemical properties, microbial community structure and function and the abundance of pathogens. Preliminary results suggest that short-fallow periods can improve soil quality in fields where ginseng is cultivated.

## Materials & Methods

### Experimental site

Three samples were collected in Erdaogang (41°35′56.99″N, 128°2′42.55″E), which is a village in a mountainous region in Malugou, Changbai County, Baishan City, Jilin Province, China. The average annual temperature is 2.0 °C, and the mean annual precipitation is 691 mm. The frost-free period is approximately 90–135 d, and the average yearly sun exposure is 2,466 h. The lowest temperature period from December to February of the next year is −17 °C on average, and the lowest temperature is −20 °C in January. Frost appears at night and thaws during the day in the middle of October. In early November, the frost layer deepens gradually, and the thickness of the frost layer reaches between 150 cm and 200 cm in February. The ground begins to thaw in the middle of March, and thaws completely by the middle of May. The depth of frozen soil is directly related to humidity, and snow depth. The soil is classified as dark brown forest soil, a typical subcategory of dark brown soil with six typical characteristic layers: AOO (leaf litter layer), AO (dark semi-decomposed organic matter layer), A1 (humus layer), Ab (transition boundary layer), B (illuvial layer), and C (parent material layer).

### Experimental design

The field experiment was conducted in October 2014. Three kinds of sampling fields were chosen: the field identified as “fallow field” (Hx2) was the field where ginseng had been cultivated for two to four years, harvested, and then the field was left fallow for two years. Another sampling field was designated the “ginseng cultivated field after fallow” (Clsd); this was the field where ginseng had been cultivated for two to four years, was harvested, and ginseng was cultivated for two years. A forest field (Hx0ks) with trees (*Betula platyphylla*, *Larix gmelinii, etc.*) was chosen as the control.

Each plot was 5 × 10 m^2^, and ginseng sampling fields were managed using the same agronomic practices. Hx2 and Clsd were old ginseng fields. The distance between the two treatments plot was 50 m, and the control forest soil plots were located next to the Hx2 and Clsd plots.

### Short-term fallow procedure of cultivated ginseng field

When ginseng roots had been harvested in the fall of 2014, chosen field plots (Hx2) were irrigated and the soil water content was increased up to about 50–60% of the maximum field water capacity. The soil was tilled and furrowed with a rotary tiller and 30 g of dazomet fine granules (98% C_5_H_10_N_2_S_2_) per square metre was spread. The soil was sealed with plastic film for more than 20 days, then opened for 15 days, so that the dazomet agent could completely evaporate. A total of 500 g per square meter of forest leaf fertilizer was broadcast into the soil, which marked the start of two years of fallow.

### Ginseng variety and soil samples collecting

We used the ginseng variety, Damaya (*Panax ginseng* strains Damaya). Ginseng seedlings were replanted in 2014 in the plot (Clsd) and were managed using the same conditions mentioned above. This marked the start of two years of ginseng growth.

Soil samples (depth: 0–20 cm) were collected with a four-point sampling method to analyze soil nutrients and microbial community structure. Different treatment soils were randomly collected from five sampling sites in each plot in October 2016. Soil samples were sealed in plastic bags and stored in dry ice before being transported to the laboratory. All soil samples were divided into two fractions: the soil samples, mixed from five sampling sites in each sample plot with stones and large roots removed, were ground through a two mm sieve to measure soil nutrients; another fraction of the soil samples were stored at −80 °C for high-throughput sequencing to analyze the soil bacterial and fungal community structure and diversity.

### Soil nutrient determination

The amount of organic matter was determined by quantifying the amount of oxidized soil carbon based on its reaction to acidic dichromate (Cr_2_${\mathrm{O}}_{7}^{2-}$) (Zhang et al., 2012). Soil available nitrogen was determined by alkaline hydrolysis diffusion method (Zhou et al., 2020). Total nitrogen was determined using the Kjeldahl determination (Lu & Foo, 2000). Available phosphate (AP) was extracted with NaHCO_3_, and was then determined by molybdenum antimony spectrophotometry (UV-1800 spectrophotometer; Agilent, Santa Clara, CA, USA) at 880 nm, based on the National Environmental Protection Standards of the People’s Republic of China (HJ 704-2014). The C:N was calculated based on the ratio of soil organic carbon (SOC) of total nitrogen, and SOC was determined by the K_2_Cr_2_O_7_ redox titration method (Nelson & Sommers, 1983).

### DNA extraction and high-throughput sequencing

Approximately 0.5 g of the chilled soil samples were used to extract the total genomic DNA using a Fast DNA SPIN Kit (MP Biomedicals, Santa Ana, CA, USA) according to the manufacturer’s instructions. Data were collected as previously described in Miao et al. (2022). Specifically, in PCR amplification, the primers (5′-ACTCCTACGGGAGGCAGCA-3′) and (5′-GGACTACHVGGGTWTCTAAT-3′) were used to amplify the V3–V4 of the 16S rRNA gene for bacterial community analysis. Primers ITS1F (5′-CTTGGTCATTTAGAGGAAGTAA- 3′) and ITS1R (5′-GCTGCGTTCTTCATCGATGC-3) were used to amplify the ITS region for fungal community analysis. The PCR reaction was run using Eppendorf Mastercycler^®^ Pro S thermal cycler as follows: initial denaturation at 98 °C for 2 min, 10 s denaturation at 98 °C for 40 cycles, annealing at 60 °C for 30 s, and extension at 72 °C for 1 min, and finally at extend at 72 °C for 10 min. PCR products were purified using a Qiagen PCR Purification Kit (Qiagen, Inc., Shanghai, China) and were pooled in equimolar concentrations. High-throughput sequencing was performed using an Illumina MiSeq platform (Biomarker Technologies Co. Ltd., Beijing, China).

### Data analysis

The Quantitative Insights into Microbial Ecology (QIIME 1.8.0) toolkit was used to analyze the sequences. After removing low-quality or ambiguous sequences, FLASH (Fast Length Adjustment of Short reads) (v1.2.7) was used to select high-quality, paired-end reads (Magoč & Salzberg, 2011).

OTUs were defined as similarity between sequences higher than 97%. Based on the results of OTU analysis, taxonomic analysis was performed on the samples at each classification level to obtain the community structure diagram of each sample at the taxonomic level of phylum, class, order, family, genus and species. QIIME was used to calculate the *α* diversity index, including the Simpson index, Chao1 index, ACE index and the Shannon index, and to draw the sample dilution curve. PICRUSt software (Yin & Wang, 2021) was used to predict functional gene composition from 16S rRNA sequences, and metabolic pathways of functional genes were observed through the KEGG database (Palù et al., 2022), leading to heat map using origin software (Biomarker Technologies Co. Ltd., Beijing, China).

## Results

### Soil nutrient content

Soil organic matter (SOM), total N, available N, available P, and C:N ratios were higher in the sample collected from the fallow plot (Hx2) and the forest plot (Hx0ks) as compared to the sample collected from the plot that was returned to cultivation (Clsd) (Table 1). Soil nutrient content were similar for the fallow and forest plots.

**Table 1:** Nutrient content of forest soil (Hx0ks), two years fallow soil (Hx2) and replanting soil after fallow for two years (Clsd).

Sample	SOM (g/kg)	TN (g/kg)	AN (mg/kg)	AP (mg/kg)	C/N ratio
Hx0ks	118	4.59	296	16.4	14.9
Hx2	100	4.28	294	15.2	13.5
Clsd	87	4.15	238	13.8	12.5

**Notes.**

SOM soil organic matter TN total nitrogen AN available nitrogen AP available phosphorus

### Basic information on the sequencing data

**Figure 1:**
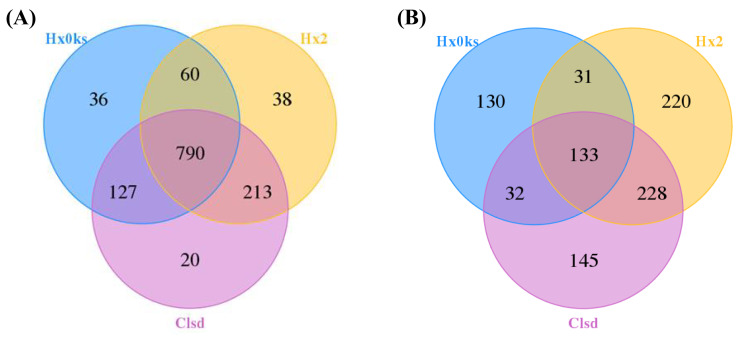
Distribution of OTU numbers of bacteria (A) and fungi (B) in Hx0ks, Hx2 and Clsd. Hx0ks, Hx2 and Clsd, respectively, represent the forest soil, two years fallow soil, and the replanting soil after fallow for two years.

A total of 390,753 bacterial 16S rRNA and 320,897 fungal ITS gene sequences passed the quality control tests. 16S rRNA and ITS gene sequences of each sample ranged from 64,542–65,311 and 52,718–58,334, respectively. After optimized sequence clustering, the number of OTUs of bacteria and fungi in the soil were 1,013–1,150 and 326–612, respectively (Table 2). The number of bacteria unique to libraries generated from the fallow or the forest plot was more than the number of bacteria unique to the plot (Clsd) that was returned to cultivation (Fig. 1A). The number of fungi unique to a specific plot, was highest for the fallow plot (Hx2) (Fig. 1B).

**Table 2:** The diversity and richness index of bacteria and fungi in forest soil (Hx0ks), two years fallow soil (Hx2), and replanting soil after fallow for two years (Clsd).

Sample	Number of bacterial OTUs	Simpson	Chao 1	ACE	Shannon	Number of fungal OTUs	Simpson	Chao 1	ACE	Shannon
Hx0ks	1,013	0.0089	1,100	1,065	5.76	326	0.0647	376	360	3.51
Hx2	1,101	0.0057	1,185	1,157	6.04	612	0.0495	635	632	4.42
Clsd	1,150	0.0081	1,212	1,189	5.89	538	0.1406	591	554	3.80

**Figure 2:**
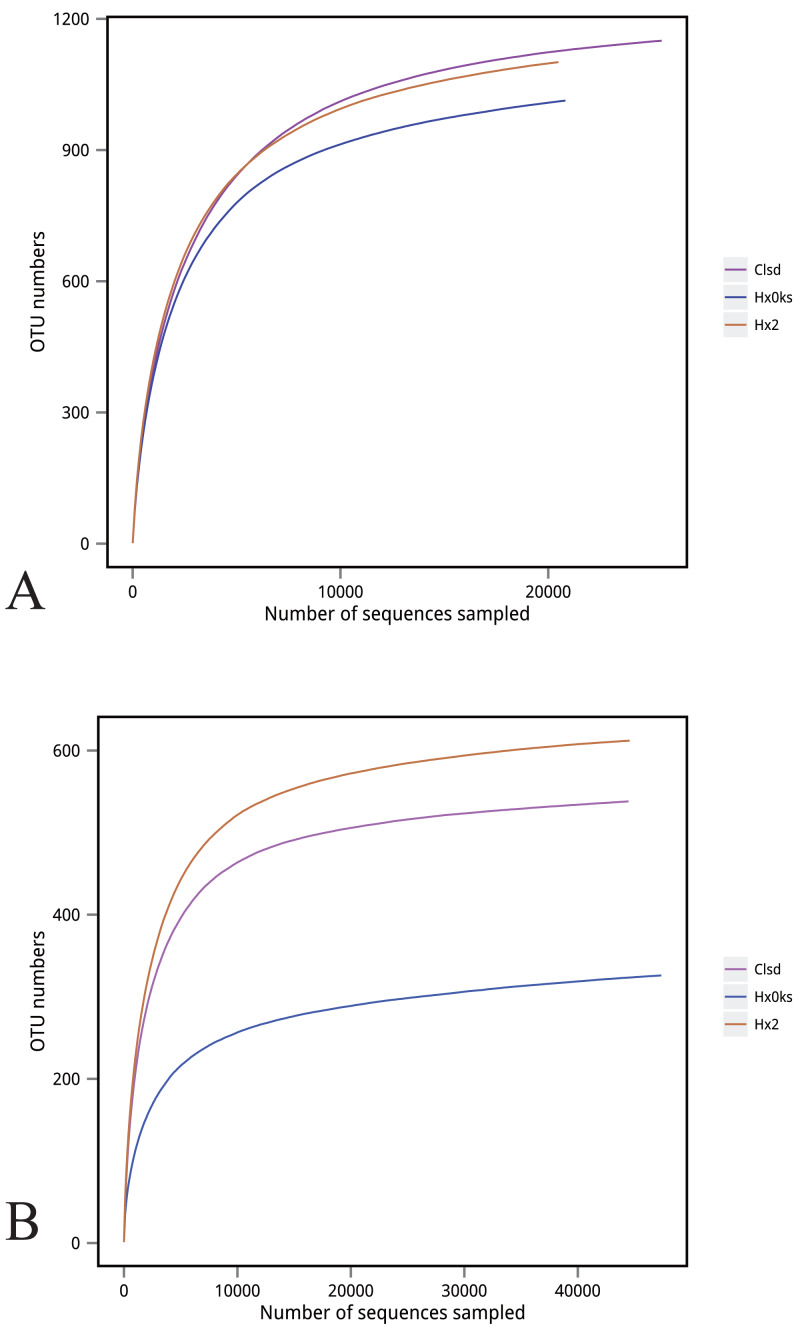
Rarefaction curve of bacteria (A) and fungi (B).

The slope of the rarefaction curve was flat under different similar cut-off values, indicating that this sample of sequences were sufficient for data analysis (Fig. 2).

### Microbial community diversity analysis

The library generated from a soil sample collected after two years of fallow period showed higher alpha diversity of bacteria and fungi communities in soil relative to plots that remained in cultivation (Table 2).

### Changes in the relative abundance of bacterial communities

**Figure 3:**
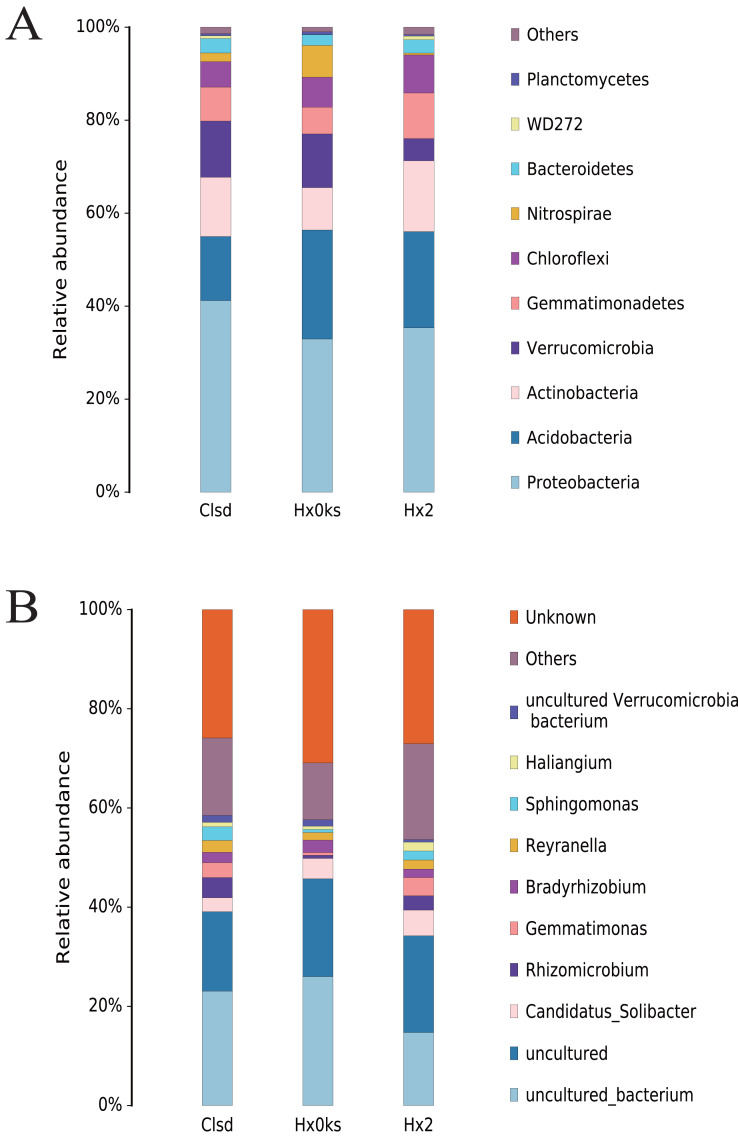
The composition of bacterial communities in forest soil (Hx0ks), two years fallow soil (Hx2) and the replanting soil after fallow for two years (Clsd).

Cultivation did not significantly change the structure of bacterial communities, at the phylum level, in soil (Fig. 3A). The main phyla detected (Acidobacteria, Proteobacteria, Actinobacteria, Verrucomicrobia and Gemmatimonadetes) were the same in the three samples (Fig. 3A). Among these, Acidobacteria and Proteobacteria accounted for the majority of bacteria observed. Similarly, the relative abundance of bacterial genera was similar between the three libraries but these genera were not well characterized. About 80% of OTUs were either unknown genera, uncultured or others (Fig. 3B).

### Changes in the relative abundance of fungal communities

**Figure 4:**
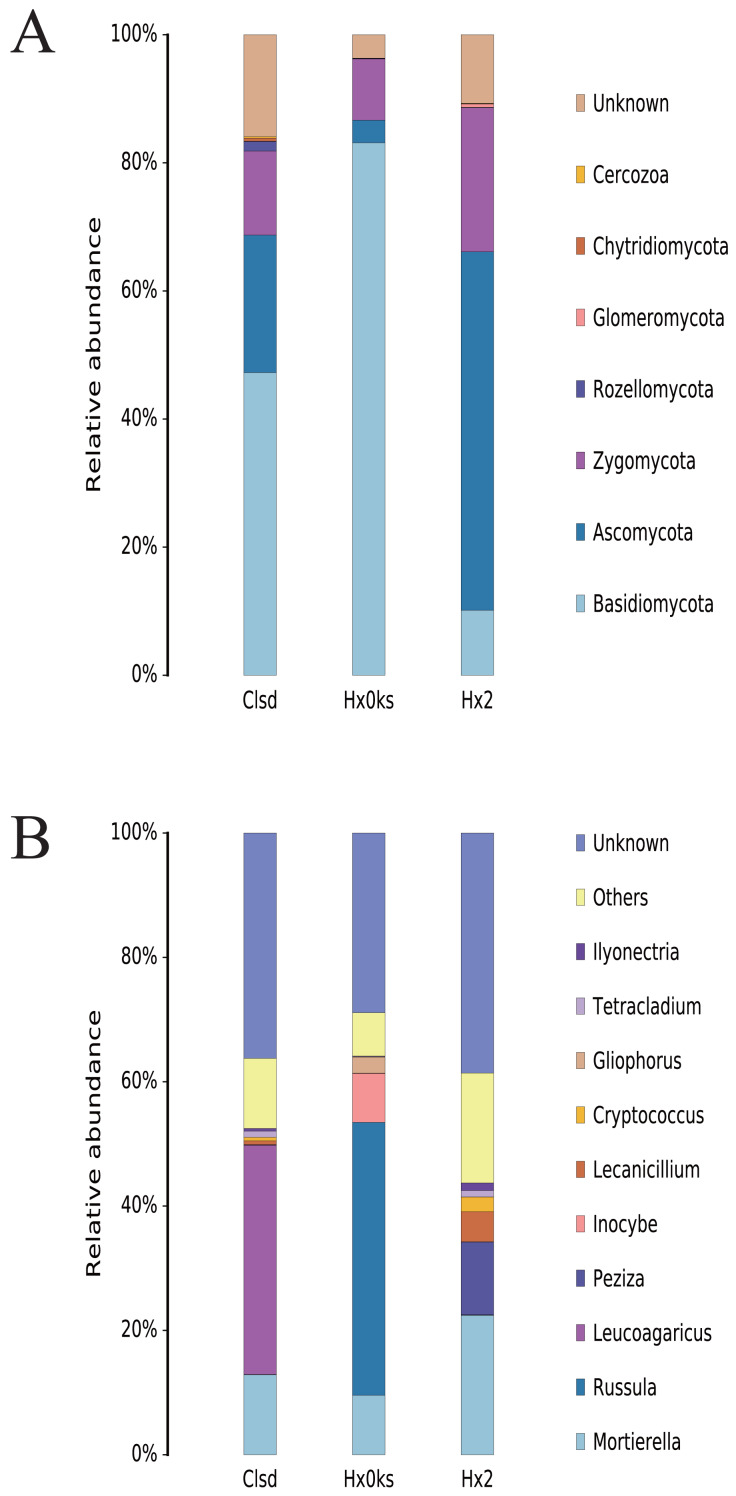
The composition of fungal community’s phyla (A) and genus (B) levels in forest soil (Hxoks); two years fallow soil (Hx2) and the replanted soil after fallow for two years (Clsd).

The main fungal phyla detected in the three samples were Basidiomycota, Ascomycota, and Zygomycota; however, each sample had a partially unknown sequence at the phyla and genus level due to the limited resolution of ITS sequencing (Fig. 4A). Basidiomycota and Ascomycota accounted for >70% of the fungal phyla detected. In the library generated from the forest sample (Hx0ks), *Russula* (44%) was the dominant fungal genera identified (Fig. 4B). In the library generated from the fallow and cultivated field samples, *Mortierella* and *Leucoagaricus* respectively were the dominant fungal genera identified.

### Function prediction

**Figure 5:**
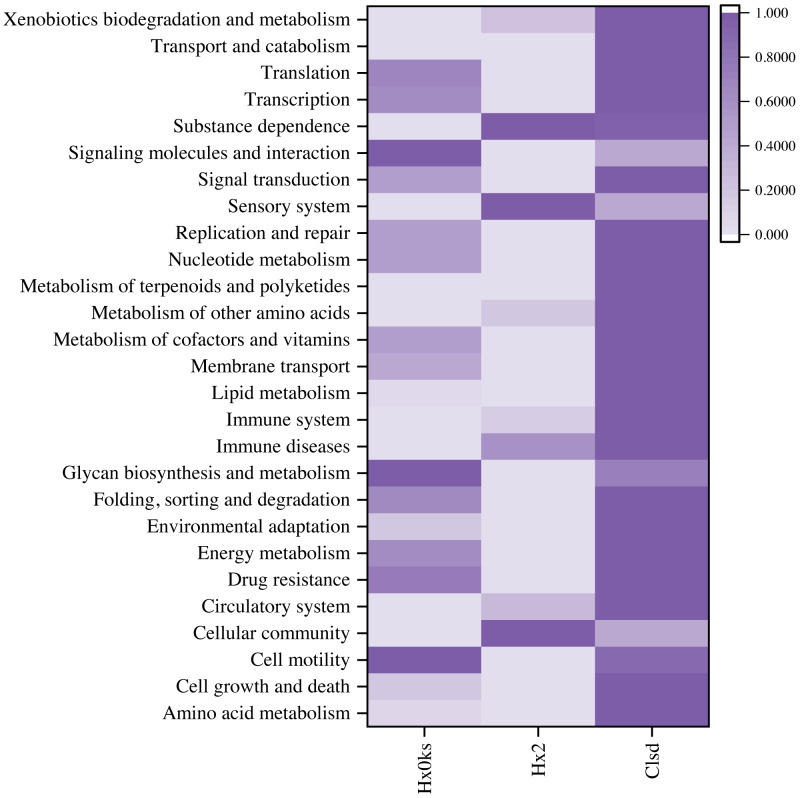
Prediction of microbial community function using a heatmap.

Differential analysis of KEGG metabolic pathways allowed for the identification of approximately 27 intensive metabolic pathways (Fig. 5). The sample from a forest plot showed many pathways for signaling molecules and interaction, glycan biosynthesis and metabolism, cell motility, drug resistance, folding, sorting and degradation, and energy metabolism. The sample a fallow plot showed many pathways for substance dependence, sensory system, immune diseases, circulatory system, and cellular community. The sample from the cultivated plot showed pathways for xenobiotics biodegradation and metabolism, transport and catabolism, signal transduction, nucleotide metabolism, metabolism of terpenoids and polyketides, environmental adaptation, drug resistance, cell growth and death, amino acid metabolism. Compared to the sample from forest soil, pathways of signaling molecules and interaction, glycan biosynthesis and metabolism, and cell motility were reduced.

### Changes in the relative abundance of pathogens

**Figure 6:**
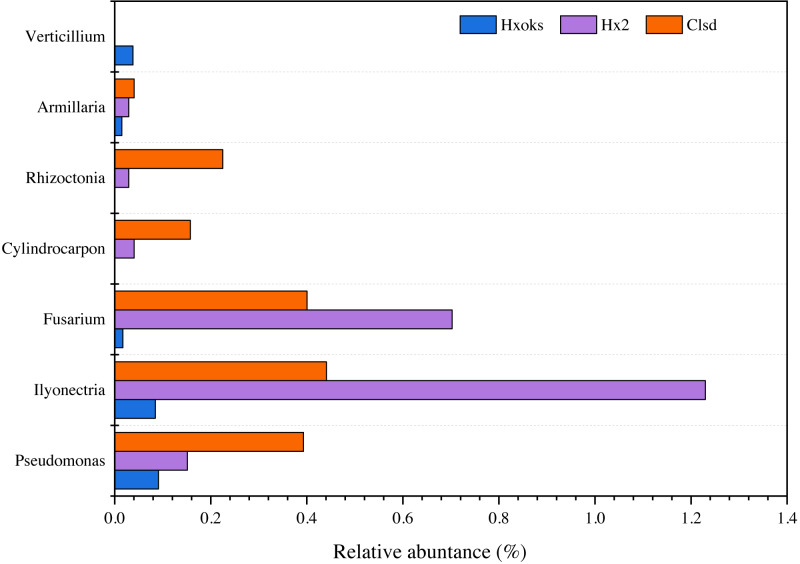
Relative abundance of major ginseng pathogens in Hx0ks, Hx2 and Clsd at the genus level.

Fallow periods appeared to reduce the relative abundance of fungal pathogens of ginseng. Pathogens were relatively rare in the library generated from the forest soil sample (Fig. 6). The relative abundance of *Armillaria*, *Rhizoctonia*, and *Cylindrocarpon*, were lower in the fallow plot (Hx2) than the cultivated plot; however, *Fusarium* (0.70%) and *Ilyonectria* (1.22%) were relatively higher in the library generated from the fallow plot (Fig. 6).

Compared to the fallow field, the relative abundance of *Armillaria* (0.04%), *Rhizoctonia* (0.22%), *Cylindrocarpon* (0.15%), and *Pseudomonas* (0.39%) was higher in the library generated from the cultivated plot.

## Discussion

This study preliminarily assessed the differences in microbial diversity and the relative abundance of pathogens between forest soil, two-year fallow soil, and replanting soil after fallow for two years. Fallow management improved soil nutrients in both topsoil and upper soil compared to replanted soil, which might explain the higher diversity of microbial communities in soil that had not been replanted for two years. In addition, the relative abundance of pathogenic fungi in the soil was reduced in the two years of fallow replanting. This suggests that soil fallow can effectively heal arable land and solve agro-ecological problems, such as reduced biodiversity, reduced soil fertility, which are associated with tillage (Li et al., 2018). This is consistent with the observation that extension of the fallow period, decreases the abundance of pathogens in fallow land, and species evenness increases significantly (Bao et al., 2020), but contrasts with Li et al. (2019), who found that short-term fallow did not significantly alter the richness and diversity of fungal and bacterial communities. Stoichiometric changes in C:N ratios affect biogeochemical cycles in terrestrial ecosystems through ecosystem productivity and degradation processes (Sardans, Rivas-Ubach & Peñuelas, 2012). In this study, C:N ratios in soil replanted after two years of fallow was reduced (Table 1). We found support in the literature for this result (Zhang et al., 2022). Organic matter provides nutrients and a living environment for soil microorganisms, while phosphorus is an essential nutrient for plant growth and plays an important role in the growth of ginseng and in improving resistance to pathogens (Johnston, Poulton & Coleman, 2009; Li, 1995). Ji et al. (2021) showed that microbial communities in ginseng-grown soil were very sensitive to environmental factors, with organic matter, effective phosphorus and potassium driving the composition of fungal communities.

Replanting degrades soil physicochemical properties, changes the soil microbial community and can lead to outbreaks of plant diseases (Ogweno & Yu, 2006; Wu et al., 2008). Among these, changes in the soil microbial community are the main factor affecting crop replantation (Manici et al., 2013; Nayyar et al., 2009). Our results are consistent with the observation that soil fallow periods significantly altered the relative abundance of some genera of bacteria and fungi (Tian et al., 2017); however, without replication our results must be considered preliminary.

Analysis of the bacterial community composition showed that the dominant bacterial genera in the forest soil were *Candidatus Solibacter* (4%) and *Bradyrhizobium* (2%) (Fig. 3B). *Candidatus Solibacter* has the ability to utilize complex substrates such as chitin, hemicellulose, pectin, starch and xylan (Ward et al., 2009). In addition, *Bradyrhizobium* can secrete extracellular polymers (EPS) in response to environmental stresses such as pH and temperature changes, shortages of carbon sources and toxic components (Belova, Pankratov & Dedysh, 2006; Quelas et al., 2016). *Rhizomicrobium* (4%) and *Gemmatimonas* (3%) dominated in the two-year fallow replanted soil. *Gemmatimonas* dissolves insoluble substances and can improve the plant’s ability to withstand adversity or produce antibiotics to protect it from disease in the event of a fungal attack (Dong et al., 2018a).

For the fungal community, the relative abundance of the genus *Russula* was significantly higher in the forest soil than the plots that had been cultivated (Fig. 4B). *Russula* plays an important role in the global forest ecosystem, as its mycelial network facilitates the uptake of nutrients in higher plants and its entities contain bioactive compounds with antimicrobial and antioxidant properties (Li et al., 2018). The relative abundance of *Peziza* was also significantly higher in the two-year fallow soil than in the forest soil and the two-year fallow replanted soil. The genus *Peziza* is found in all soil types and most species of *Peziza* prefer environments with a low organic matter content and alkalinity (Barseghyan & Solomon, 2011). In contrast, the relative abundance of *Leucoagaricus* was significantly higher than that of other fungi in the two-year fallow replanted soil. The genus *Leucoagaricus* produces laccase and co-oxidative enzymes that play an important role in the degradation of plant residues and provide plant biomass for soil-borne post-zooplankton (Aylward et al., 2013).

Soil in which ginseng has previously been cultivated was different from bulk soil in terms of genetic diversity and ecological function (Li, Ying & Ding, 2014). Ying et al. (2012) also found that microbial communities and their metabolic profiles exhibited significant differences during ginseng cultivation. These differences may reflect the age of the ginseng and the quantity of root exudates released by the ginseng. According to the ginseng disease species of each country recorded in the ginseng root disease survey study in Jilin Province (Chen et al., 1964), six fungal pathogens (*Cylindrocarpon*, *Rhizoctonia*, *Armillaria*, *Verticillium*, *Ilyonectria* and *Fusarium*) and one bacterial pathogen (*Pseudomonas*) were identified in this article. When comparing the relative abundance of *Ilyonectria* and *Fusarium* in the two-year fallow and two-year fallow replanted soil, the relative abundance of *Ilyonectria* and *Fusarium* in the two-year fallow replanted soil were 0.78% and 0.30% lower than the two-year fallow soil, respectively (Fig. 6). Root rot, caused by *Ilyonectria*, is the most serious chronic ginseng disease and occurs in all ginseng growing regions (Guan et al., 2020). Root rot caused by *Fusarium* can lead to the death of young seedlings and the decay of large numbers of ginseng roots, and which is easily spread by the release of conidia (Tian et al., 2018). In this study, planting density might be a critical factor influencing the reduced abundance of pathogens in the replanted soil. Ginseng seedling emergence is heavily influenced by sowing density, as are root diameter and root weight (Suh et al., 2018). Sowing densities of approximately 33–42 seeds/m^2^ are ideal for greenhouse direct seeding cultivation to obtain optimal yields of 3-year old ginseng. Alternatively, reduction in the relative abundance of these two pathogens in the two-year fallow replanted soil may be due to the accumulation of inter-root secretions and root rafter cells. Root secretions can attract beneficial microorganisms and influence the accumulation of inter-rooted microflora, thus increasing the plant’s ability to adapt to its environment (Ren et al., 2021). In addition, shed root cells can still be viable and the percentage of viable cells can reach 90%. These root cells have an attractive, repulsive, and inhibitory effect on pathogenic bacteria and fungi and can secrete anti-microbial compounds to inhibit pathogens (Santoyo et al., 2021).

## Conclusion

The low relative abundance of pathogenic fungi *Ilyonectria* and *Fusarium* in the two-year fallow replanted soil is consistent with an altered structure and function of the soil microbial community. However, these results are preliminary. Replicated plots are needed to investigate the role of fallow rotation.

##  Supplemental Information

10.7717/peerj.14758/supp-1 Supplemental Information 1 Raw data of bacterial community of Clsd samples

10.7717/peerj.14758/supp-2 Supplemental Information 2 Raw data of fungal community of Clsd sample

10.7717/peerj.14758/supp-3 Supplemental Information 3 Raw data of bacterial community of Hx0ks samples

10.7717/peerj.14758/supp-4 Supplemental Information 4 Raw data of bacterial community of Hx2 samples

10.7717/peerj.14758/supp-5 Supplemental Information 5 Raw data of fungal community of Hx2 samples

10.7717/peerj.14758/supp-6 Supplemental Information 6 Raw data of fungal community of Hx0ks samples

## Additional Information and Declarations

### DNA Deposition

The following information was supplied regarding the deposition of DNA sequences: 16S and 18S rRNA sequences of soil described here are available at GenBank: SRR17818411, SRR17818412, SRR17818413, SRR17818414, SRR17818415 and SRR17818416.

### Data Availability

The following information was supplied regarding data availability: The raw measurements are available in the Supplemental Files.
